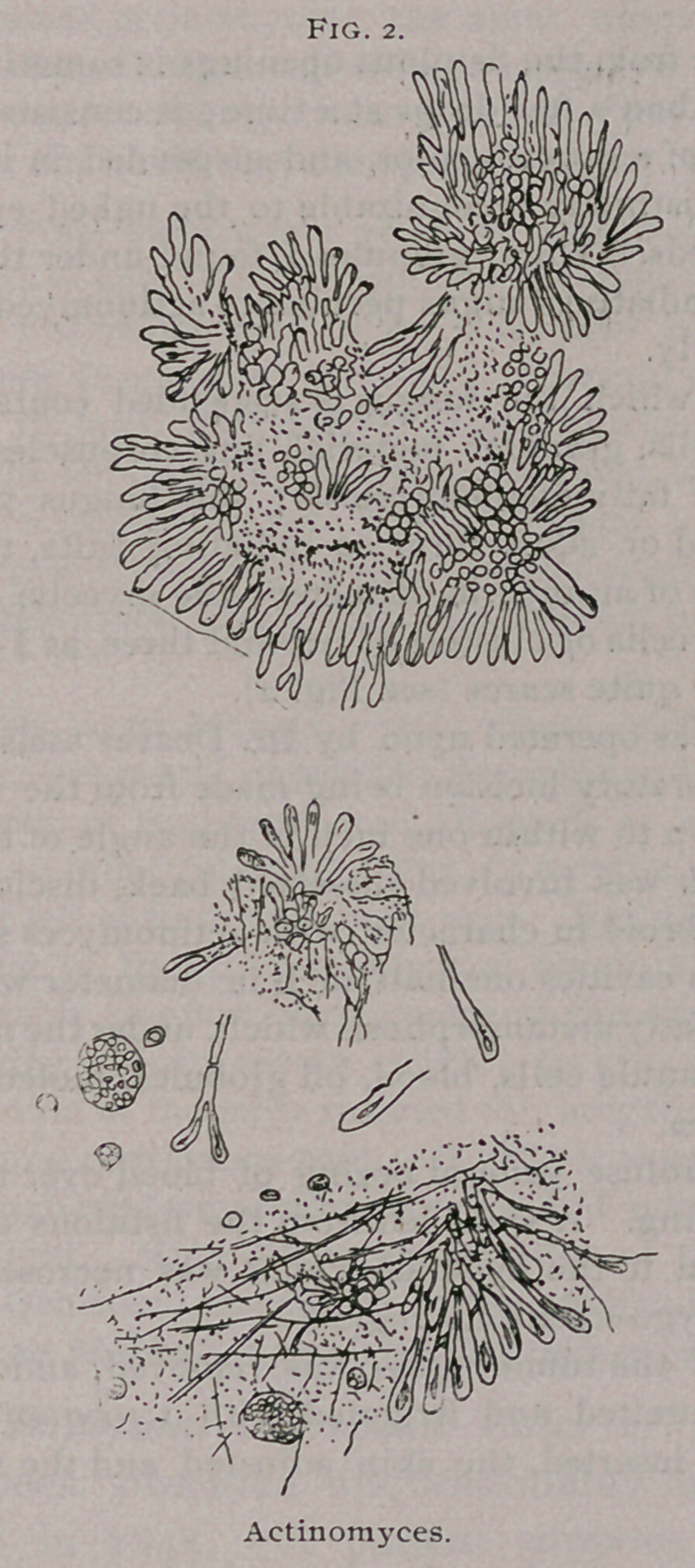# The Pathology of Actinomycosis, with Record of Cases and Experiments

**Published:** 1889-07

**Authors:** George A. Bodamer

**Affiliations:** Physician-in-Chief to the German Hospital, Philadelphia; Late Assistant to the Pathological Laboratory, University of Pennsylvania


THE JOURNAL

— OF —

COMPARATIVE MEDICINE AND SURGERY.

VOL. X. JULY, 1889. NO. 3.

### ORIGINAL COMMUNICATIONS.

### ART. XII.—THE PATHOLOGY OF ACTINOMYCOSIS, WITH RECORD OF CASES AND EXPERIMENTS.

BY GEORGE A BODAMER, M.D., B.S.,

*Physician-in-Chief to the German Hospital, Philadelphia; Late Assistant to the Pathological Laboratory, University of Pennsylvania.*

[Concluded from page 126.]

*Morphology of the Actinomyces.*—This fungus was discovered by Professor Bollinger in 1877 in a tumor from the jaw of an ox, and Dr. Harz, Professor of Botany at the Munich Veterinary School, gave the above name on account of the radiating arrangement of its mycelia. There is, however, evidence that Langenbeck in 1845 had seen and described a fungus similar to the Actinomyces, which he found in a case of necroses of bone in man. The morphology of this fungus has been also well treated of in the writings of J. Israel, Ponfick, Johne, G. Fleming, O. Israel, and others. (See Bibliography.)

It is sufficient here to say that the Actinomyces is a large mould fungus (Hypho-mycetis), closely allied to the ordinary ubiquitous green mould, *Pennicillium glaucum*, but differing from it, and, in fact, from all other known moulds in the radiating arrangement of the mycelia, as seen in fully developed tufts.

The individual adult plant consists of a round or oblong tuft of club-shaped mycelia, arranged somewhat after the manner of a bunch of grapes, except that the mycelia or bypheus of the fungus are closely packed and radiate from one central root-cell in a star-shaped manner.

In its undeveloped state, in which it often persists, the Actinomyces presents a single club-shaped mycelia, or in the form of conical figures of two or three mycelia springing from one root-cell, and presenting, as it were, abortive individuals of the fungus.

The majority of these tufts are aggregated in mulberry-like masses of from 0.5 to 1 millimetre in diameter, and appear to the unaided eye as very minute dull-white or yellowish granules. Very frequently the tufts are somewhat calcified, and then it is difficult to make out their composition. By slight pressure made upon it the fungus tufts are considerably altered in appearance, and mostly assume the shape of a spheroidal segment, wherein some of the organisms can be distinctly traced thoughout.

The Actinomyces is the only fungus belonging to the class of moulds that have been found in the interior of animal tissues, such as the bones, and occurring also in the metastatic deposits in the lungs and elsewhere.

It might be said in general that there is no difficulty in detecting the fungus, on account of its large size and peculiar shape. Staining with aniline dyes is, however, advantageous, as the Actinomyces retain the aniline dyes about as well as the tubercle bacilli. I found it most advantageous to use the bacillus dye for specimens to be demonstrated.

*Detection of the Fungus in Matter other than Actinomycosis.*—1st. Actinomyces in necrosis of teeth in man. I discovered accidentally the Actinomyces in the pus from an abscess of the alveolar process of the superior maxilla. A specimen of pus was examined on many occasions from a necrosed tooth of this locality. The man upon whom the observation was made had, on account of annoyance from gum boil, a hole drilled through the gums, superior maxilla, into the root of the tooth, which was necrosed, and this was discharging pus at intervals for at least two or three years. A preparation of this pus showed, on repeated occasions, a very distinct radiating fungus, which was also seen by Dr. Formad, and which we fully identified with the Actinomyces.

2d. Actinomyces was also observed by me twice in pus from necrosis of left sub-maxillary bone in a woman who was suffering from that affection. The fungus did not appear fully developed in either of these two cases, but when put into culture, well developed typical tufts were seen.

3d. I detected the Actinomyces in the scrapings from tongue, tonsils and mucous membranes of mouth in a number of cattle. Microscopical examinations were made of fifteen head of cattle, including oxen and cows. I made daily examinations, selecting a time of from twelve to twenty-four hours after they had been fed, expecting by this time to allow the mouth to act as a culture soil for the fungi that might be retained between the teeth or adhere to the mucous membrane of the mouth and tonsils. In five out of the above mentioned fifteen animals very striking examples of the Actinomyces were discovered.

The fungi were found mostly adherent to particles of straw, chaff or corn adhering to the mucous membrane or sticking between the teeth.

4th. I found Actinomyces in particles of animal food.

(*a*) Bran, or the outer coats of grain, revealed the fungus in considerable quantity. An infusion of bran was made and examined microscopically a few hours after standing, when several small fungoid bodies, represented in Fig. 2, were seen. At first not much stress was laid upon this, but subsequent examinations fully revealed their identity with the Actinomyces. Repeated examinations of these infusions were made daily, and every now and then fragments of the fungus were detected, and where care was practised in making and cultivating these infusions fully developed Actinomyces were occasionally observed.

(*b*) Examinations of the siftings of grain. Small fragments of grain were taken from the bottom of a large chest in which grain was kept in a barn (corn mostly) and infusions were made of this material. In these infusions I found several times distinct fragments of the Actinomyces as represented in Fig. 2. Examinations were kept up daily for two or three weeks occasionally with positive, more often, however, with negative results.

(*c*) Examination of chaff or husks of grain also revealed the fungus. Of these husks infusions were made as before. I observed on several occasions large radiating masses of the Actinomyces and fragments of crushed tufts in all forms and shapes were frequently found. Most satisfactory results were obtained by agitating the dry chaff in a glass bottle, and adding to a small portion of this a drop of distilled water.

(*d*) In smut from corn the fungus was found, but in some cases I could not positively identify the Actinomyces. I made cultures of this, but the results were doubtful.

(*e*) Fodder and pith of corn stalk were examined, together with scrapings from the outside of the stalk. Small pieces of these were shaken up in distilled water and immediately examined. I saw fungoid bodies in this infusion that I could not tell from Actinomyces, and believe they were the fungus in question.

Most striking specimens were seen whenever the fodder was moistened and allowed in a moist chamber to partly undergo decomposition.

The common occurrence of the Actinomyces in the food of animals, as proven by my examinations, suggests that this fungus is only a concomitant in Actinomycosis, and further suggests that the fungus should be looked for in the lesions of other animal ailments.

*The Isolation and Cultivation of the Actinomyces.*—Cultures were made in strict pursuance with the usual well known methods in Bacteriology in a well-equipped laboratory. All known culture soils, but principally potatoes—pepton-gelatine, agar-agar and blood-serum were repeatedly tried, but only the latter coagulated in the test-tubes found available and useful for raising good crops. This I believe was also the experience of O. Israel, in Berlin.

The chief objection to all *other media* outside of blood-serum appeared to be that ordinary moulds and bacteria grow better in them than the Actinomyces.

In the first generation the fungus did not appear to grow well, but in the second the crop proved successful. Some of the cultures I carried to the fourth generation which appears to be the limit of prosperous growth in artificial culture for the Actinomyces. Beyond the fourth generation, *e. g.*, in the fifth or sixth I usually failed to obtain a good crop of the fungus. The best crops were obtained in the second and fourth generations where the fungus appears to reach the perfect size and development or the stage of tufts. In the third and fourth generations the stage of tufts was less often reached than in the second, and the fungus occurs only in radiating fragments with imperfect conidiæ.

When these imperfect conidiæ were, however, inoculated into an animal they usually developed into perfect tufts.

The fungus as I observed from many trials grows best to its full development in blood-serum at the bodily temperature, or at least transforms at this temperature into those radiating clusters which we are accustomed to see in the lesion of Actinomycosis.

The growth of each generation took one week on the average; further than one week the fungi would not grow well unless transplanted to fresh culture soil.

The fungus in culture appears to the naked eye in successful cultures, as a yellow or grayish-yellow film closely resembling lycopodium, which in growth keeps at the surface as long as it is pure and not contaminated. This appearance of the fungus enables one to recognize it promptly by the unaided eye. It does not liquify the serum. If liquifaction or mould of any other color or form than above stated appear, it shows that the culture is impure.

*Actinomycosis in Man, with Report of Cases.*—Although about forty-nine cases of actinomycosis in man have been recorded abroad, but four authenticated cases are found in American medical literature; these cases are two by Dr. Murphy, of Chicago (*N. Y. Medical Journal*, 1885, vol. xli. pp. 17–19); one by Dr. Schirmer (*Chicago Med. Journal and Examiner*, vol. liii. p. 354); and the last by Dr. Ochsner (*Ibid.*, vol. liii pp. 1–3); to be quoted below. Hence I think the case which came under my observation worthy of record.

Wm. M. H., aged thirty-two years. Born in England; occupation miner; mother, four brothers, and three sisters living in good health; father died of senile debility. Patient had ordinary diseases of childhood, besides pleurisy at the age of twenty-eight years. He came to this country at the age of twenty-one years, and continued his occupation steadily as a miner, and he was often subjected to injuries of the head. In 1877 he was severely struck on the right side of the head by a prop. He continued to work in the mines, and while in the Stockton Coal Company's mines, Luzerne Co., Pa., he noticed for the first time a swelling at the angle of the right inferior maxillary bone, which would go and come; and, as the patient expressed it, came to stay in the winter of 1882, and soon after reached the size of a chicken's egg, when he became alarmed, and began poulticing it; at the same time he had the three upper and three lower back molars of the right side drawn. All six teeth were sound.

Patient states he was advised to keep the gums bleeding and irritated, believing this treatment would reduce the swelling and effect a cure. A growth, however, promptly made its appearance, filling up the cavity made by the extraction of the teeth, of the lower and upper jaws. About this time he ran against a projecting drill (an iron rod six feet in length with a sharp, square end two inches broad), which severely injured the tumor; the wound bled profusely but healed promptly.

During 1883 the swelling made little progress, and in May, 1884, he came to Philadelphia, and was admitted to the surgical wards of the Pennsylvania Hospital, where, as he states, a tumor was removed. He then returned to work, and had no medical treatment for four years, when he again

FIG. 1.

Case of Actinomycosis.

entered the Pennsylvania Hospital in November, 1888, having in the meantime sustained some more injuries of the diseased jaw. There is no evidence that a diagnosis of actinomycosis had been made.

On Jan. 11, 1889, he came under my observation at the German Hospital[1] when I examined him in conjunction with Drs. John B. Deaver and F. Gross.

*Present condition:* Patient is a muscular, well-nourished, robust man, of five feet five and a half inches in height, with good complexion, weighing one hundred and fifty pounds, and has a good appetite. The only lesion observed is a swelling on the right side of the head, face and neck (see Fig. 1, from a photograph) which consists of a hard, infiltrated, uniform mass extending from the clavicle to the temporal region, involving the anterior half of the temporal muscle, the muscles of the face, and the glands of the neck, and evidently in intimate union with the cranial bones of the region described. The swelling implicates also the cavity of the mouth; the buccal muscles, as well as the lower and upper jaw, are much enlarged from the swelling, which causes an ankylosis, so that the patient cannot separate his teeth more than a quarter of an inch, and is unable to take food except

FIG. 2.

Actinomyces.

by forcing it through this narrow space; this, the patient said, has been the case for the last two years. While mastication is next to impossible, the power of deglutition appears to be perfect. He uses mainly food in a crushed or liquid state.

The swelling is uniformly firm, except in the temporal region, where there is a fistulous opening; another fistulous opening is at the angle of the jaw, but numerous scars upon the neck indicate that there were a number of fistulous openings which have healed. A third fistulous opening into the mouth corresponds to the third lower molar tooth. The patient states that there had been a great deal of suppuration from the former fistulas. On the whole, the right side of the head, face, and neck is about one inch thicker than the left side; the pain is slight, somewhat increased by pressure and is more intense during the night. A large scar extending from the angle of the mouth to the lower border of the inferior maxilla indicates a former removal of some of the tumor mass in 1884, at the Pennsylvania Hospital, as the patient states.

The discharge from the fistulous openings is sometimes copious, but at present not more than a few drops at a time; it consists of a purulent, odorless, viscid liquid of a grayish color, and suspended in it are numerous yellowish granules, distinctly recognizable to the naked eye, being of the size of small poppy seeds. These granules I found under the microscope to be actinomyces, the radiating fungus peculiar to actinomycosis, and I diagnosed the case accordingly.

The fluid in which the fungus is suspended contains numerous compound granule cells, granular material, pus corpuscles, and fragments of fibres in a state of fatty metamorphosis; the fungus presents itself in its perfectly developed or adult form as radiating tufts, round or elongated, measuring $\frac{1}{200}$ to $\frac{1}{50}$ of an inch in diameter; the mycelia are unusually plain and single free mycelia or clusters of two and three, as I have seen in animal actinomycosis, are quite scarce (see Fig. 2).

The patient was operated upon by Dr. Deaver assisted by myself, Feb. 9, 1889. An exploratory incision being made from the fistulous opening in the temporal region to within one inch of the angle of the inferior maxilla, and the skin which was involved dissected back, disclosed a very vascular infiltrated mass, fibroid in character, with actinomyces studded through its entirety; also two cavities one-half inch in diameter which contained material in a state of fatty metamorphosis which, under the microscope, revealed pus, compound granule cells, blood, oil globules, molecular debris, and the fungus actinomyces.

There was a profuse general oozing of blood over the whole surface of the exposed swelling. A sinus led from the fistulous opening in the temporal region down to the zygoma, which was necrosed, and beneath the same into the pterygo-maxillary region.

A part only of the tumor mass was removed, and the exposed growth was thoroughly curetted and irrigated with 1:1000 sublimate solution; a drainage tube was inserted, the skin adjusted and the wound dressed antiseptically.

The four cases of actinomycocis in man recorded in this country previous to this case of mine are as follows:—

Case. I.—A female twenty-eight years old, suffered severe toothache of left lower jaw with subsequent swelling in mouth and throat, the swelling (abscess in left tonsil) was opened and patient recovered rapidly. About seven months later, a swelling the size of a walnut appeared on the left side of the neck, which was lanced; the pus contained actinomyces. As the swelling and induration continued to increase, an operation was performed and the growth removed. Patient made a rapid recovery, gaining twenty-six pounds in five weeks, and now (1885) is in good health.—Dr. Murphy, *New York Medical Journal*, 1885, vol. xli. p. 17.

CASE II.—Thomas C., aged eighteen, while in Ireland had severe toothache; later, a swelling appeared and enlarged with more or less pain; a carious tooth was found, a swelling was punctured, and a few drops of pus escaped which contained actinomyces; the sinus was scraped out and the parts completely healed. Later, a swelling reappeared in the same place which was opened and a drainage tube inserted, after which Dr. Murphy did not see the case, but heard that the opening healed. (Ibid.)

CASE III.—A Pole, twenty-five years of age, family history good. When eighteen years of age, while chopping wood, received a slight incised wound of cheek. Following this injury both jaws began to swell; the swelling in the left side disappeared, and that on the right side was opened and a fistula followed. In June, 1886, patient passed into Dr. Schirmer's hands. The exudate from the fistulous opening was examined by Prof. Fenger and Dr. Schirmer; and actinomyces was found. Patient lost his appetite, became emaciated, followed by severe cough and the expectoration contained actinomyces.—Dr. Schirmer, *Chicago Medical Journal and Examiner*, vol. lii. p. 354.

CASE IV.—Man, aged fifty-six years, whose occupation was stock-raising and dealing, was much exposed to draughts and cold. He suffered pain in the left antrum of Highmore, and although he had six teeth drawn which were sound, no relief followed. For six months patient suffered excruciating pains in left antrum and both eyes. Early in 1878 there was a spontaneous opening of the abscess into the pharynx, from time to time evacuating considerable pus and blood; some of the discharge entered the larynx at night giving rise to severe cough. In 1880 was operated upon, curetting and irrigation of the parts resorted to; irrigation was kept up two years, patient suffering continuous pain. In 1885–6 severe cough with dulness over lungs, roughened respiratory sounds and mucous râles followed by great loss of weight. Microscopic examination of the sputum revealed actinomyces.—Dr. Ochsner, *Journal of Amer. Medical Association*, 1886, pp. 608–610; *Chicago Medical Journal and Examiner*, vol. liii., pp. 1–3.

*The More Important European Cases of Actinomycosis in Man.*—Langenbeck observed unquestionably a case of actinomycosis in man in 1845. A patient supposed to suffer from tubercular disease was admitted to the surgical clinic of Von Langenbeck, then at Kiel. The patient had caries of the dorsal and lumbar vertebræ with some fistulæ opening in the covering skin, from which an ichorous pus containing yellow granules of the size of a poppy seed exuded; these granules were regarded as cheesy, tubercular masses. But microscopical examination made by Von

Langenbeck, and recorded at the time of examination, showed these masses to consist of fungi, the nature of which was not determined at the time, but which as it is now evident from the original drawings made at the time, was the actinomyces.

The description given leaves no doubt about the identity. The clinical feature of the case, as well as the subsequent autopsy record, confirm such presumption. The vertebræ and the surrounding soft parts presented new formations with purulent masses filled with yellow granules and other lesions fully corresponding to some of the cases described subsequently by recent observers of actinomycosis. The description of the above case of Langenbeck's was resurrected by Israel from the autopsy book of the Friedrich's Hospital at Kiel.

J. Israel (in Virchow's Archiv. Vol. 74) in 1878, or thirty-three years later, next described four cases of a new mycosis in man, identical with the case related by Langenbeck. These also were, evidently, genuine cases of actinomycosis, judging from his excellent description of the lesion and of the fungus, given by Israel, and the identity with the disease in animals as described first by Bollinger in 1877. But Israel, at the time of the publication of his cases, was not aware of this fact, and hence he and not Ponfick should be credited with the discovery of actinomycosis in man; even if he did not term the disease by its proper name. Israel's cases are so exhaustively described in Virchow's Archives, and with such precision, that I can not refrain here from giving the details.

Analysis of the cases shows that the cases referred to by Israel represent:

1st, a case of a pleural affection in a woman æt. 39 years, resembling tuberculosis, but characterized by caries of the ribs and spine, from which fistular openings led externally through the skin. The pus exuding contained the unquestionable actinomyces fungus, as may be inferred from his excellent description and drawings. This case of Israel's terminated fatally by "Chronic Pyæmia," as he says. All the lesions found post mortem correspond to actinomycosis.

2nd Case..—A man aged 36 years. This case was one of inflammation tumefactions of the alveolar processes of the lower jaw with deep-seated abscesses, fistular openings and exudations containing actinomyces granules. Recovery.

3rd Case.—A girl 9 years old, suffering from sub-periosteal abscesses at the margin of the lower jaw; coincident with caries of the third molar; from fistular openings exuded pus containing the yellow granules. Recovery.

4th Case.—In all respects analogous to case 1, and terminating also fatally.

The name by which Israel designated the fungus found, was in accordance with the suggestion of Ferdinand Cohn—Streptothrix Försteri.

Ponfick (Die Actinomycose des menschen, Berlin, 1882) describes his five cases of actinomycosis with full details. The first of these, and the one which he claims as representing the discovery of actinomycosis in man, or at least the first observation of this disease made knowingly in man, is published in the *Breslauer Aertzlicher Zeitschrift*, 1879.

This case of Ponfick's was that of a powerfully built man, aged forty-five, who had suffered from the sequelæ of Pleurisy on the left side for a year and eight months. After death there was found an extensive prævertebral phlegmonous inflammation in the posterior mediastinum, with a para-pleuritic abscess-cavity extending both to the right and left, at the level of the seventh, eighth, and ninth intercostal spaces; with this cavity there communicated a complex system of sinuses, extending through the substance of the longissimus dorsi, the scapular muscles, and the subcutaneous tissues of the whole back. The sulphur yellow fungus bodies were found upon or between the granulations of these sinuses and in their substance, as well as in the sero-purulent discharge; they were also found in a cavity of the size of a cherry, which occupied the centre of a hepatized area of the left lung (lower lobe), as well as in the exudation that filled some of the neighboring alveoli.

The second case was that of a woman aged sixty-one, admitted with an abscess of the lower part of the abdominal wall; she subsequently developed another abscess of the left iliac fossæ, without recurring symptoms of peritonitis, and died from exhaustion. After death caries (with prævertebral collection of pus) of the three lower lumbar and first sacral vertebræ, abscesses in both iliac fossæ, and perityphlitic adhesions were found. The yellow fungus bodies were discovered in the pus of the prævertebral abscess.

The third case was that of a woman, aged forty-five, who had suffered an injury of the right thumb three years before, with swelling of the arm, which did not subside, but extended to the neck and back, and was accompanied by progressive weakness. The necropsy revealed extensive sinuses on the left side of the neck and in the prævertebral tissue, a knob-like excrescence of newgrowth extending into the lumen of the internal jugular vein, a tumor of the size of an apple, growing into the right auricle and ventricle, with corresponding whitish centres in the ventricular substance, and gelatinous nodules in the spleen and in the occipital lobe of the right cerebral hemisphere. In this remarkable case the fungus bodies were found in the sinuses of the neck, in the substance of the sarcoma-like growth of the jugular vein, in the tumor of the right auricle and ventricle, and elsewhere.

In the fourth case the illness began fourteen months before death, following the extraction of an upper molar tooth ; it consisted of swelling in the region of the right maxillary joint, tumefaction of the face, and subsequently of the neck ; successive outbreaks of abscesses and sinuses in these regions. Death occurred from extreme exhaustion. The yellow fungus bodies were frequently obtained in this case from the sinuses during life. The record of the case is too elaborate to be given, even in outline ; but it may be mentioned that there was, besides the extensive sinuses and granulation-centres of the face and neck a prævertebral abscess extending from the basilar process of the occiput to the fourth dorsal vertebra, with osteophysic growth from all the bones, together with caries of both occipito-atlantal joints and of the right atlanto-axial.

In the fifth case, a boy, the first indication of illness was a year before death, when he had symptoms of pleurisy ; nine months later, there was a new and much more severe affection of the same side, with general dropsy, progressing swelling in the lower part of the back, and evacuation of pus from a cavity opposite the eleventh left rib. *Post-mortem* :—A large prævertebral cavity was found on the left side, partly retropleural at the level of the eighth, ninth, and tenth ribs, and partly retroperitoneal at the level of the last two ribs and the left kidney ; also several perforations of the diaphragm. There were numerous centres of actinomycosis in the muscles of the back, in the intercostals, and in the left psoas muscle; also in the muscular substance of the left ventricle and in the upper end of the spleen.

Four cases of actinomycosis with extensive tumefaction and deep seated abscesses with lesions having started, as usual in man, from the soft parts and subsequently proceeding to the bones, are recorded by Rosenbach, (zur kenntniss der strahlenpilzer krankungen beim menschen.) *Centralblatt für Chirurgie*, 1880, 225. Rosenbach claims that actinomycosis is a very common affection among the country people in the neighborhood of Göttingen; but that the affection is usually local and quite amenable to treatment. Further, he stated that the disease commences usually in the neighborhood of the jaws and appeared to be dependent upon carious teeth involving the dental-alveoli.

The same suggestion was also made by Ponfick and Israel. In mankind the tendency of the disease is to formation of abscesses and suppurations; in bovines there is more a tendency to tumor formation. Not having access to Rosenbach's original paper I cannot give details; but, from the brief quotation of Ponfick and Fleming the cases were fully analagous to the cases before described, and the actinomyces fungus was observed in all lesions.

Five cases of actinomycosis affecting the peritoneum and abdominal organs are reported from Vienna by Dr. Adolf Zemann,[1] assistant at the Pathological Institute there. The characteristic fungus colonies were found in all cases both in the purulent contents of the abscesses and in the tissues of the affected parts. The number of previously reported cases is small. Ponfick collected sixteen in all of his own and others, and these were published in his monograph on the subject, which appeared in 1882. These cases Ponfick divided into two groups, those which recovered and those which were fatal. The former group consisted of cases where the malady made its appearance on the jaws,—preferably on the lower jaw,—and spread downward along the neck to the clavicle.

The second group of cases comprised those in which the process announced itself by pains in the limbs, or, more generally along the spinal column and neighboring parts of the back, succeeded by pneumonia or pleuro-pneumonia of the left lower lobe.

[1] "Wien Med. Jahrbuch," 1883, page 477.

In a few cases these pains precede a prævertebral gathering which becomes a psoas abscess.

The five cases now reported seem therefore to be, not only a considerable addition to the existing observations of a but little known pathological process, but also to enlarge the clinical description of the disease. Some of these cases ran their course with fever and others without. Zemann concludes from his study of them that the important point in the diagnosis of actinomycosis is the discovery of the pathognomonic fungus. Apart from such identification the disease can neither be diagnosticated from the part affected nor from the course which it runs, since the process may be febrile and acute, or almost without fever and chronic; in which latter case it manifests itself as an induration, as a chronic inflammation of serous membrane, as an inflammation in the bones or periosteum, or as an inflammation attacking the internal organs. All of which is demonstrated by the varied points from which the fungus gains access to the body, and by almost innumerable complications attendant on its presence.

Zemann's cases certainly tend to show that by no means so simple a picture as that given in Ponfick's monograph will suffice to represent actinomycosis.

There are a few cases of actinomycosis said to be described by Kracke, Esmack, Weigert and Partsch, (Breslauer Arztliche Zeitschrift, p. 78, 1881), but I had no access to the original papers, and hence can give no details.

All the above cases of actinomycosis as seen in man have been described in Germany and by German observers. The only case described elsewhere is that of W. R. Freves, of London, Surgeon to the National Hospital for Scrofula.

Freve's (*London Lancet*, January 19, 1884), a copy of which I hereby append gives also an illustration of the external appearance of a man affected by this disease. The case is as follows:

"P. C., aged forty-five, a brick burner, was admitted on August 17th, 1883, into the Royal Sea-bathing Infirmary under my care, supposed to be suffering from a scrofulous affection of the glands of the neck. He is a powerful built, muscular man, with a good family history. His illness began sixteen months before admission with inflammation about the neck and angle of the jaw, which, however, only kept him from work some two or three days. He has lived well and been in a comfortable position for his class in life. He has never had cattle to look after. A lump near the angle of the jaw followed by inflammation, was incised. Subsequently other swellings formed. When first seen he was in fair general health and well nourished. Over the angle of the jaw and in the posterior triangle of the neck were three ulcerated and fungating surfaces, those near the angle of the jaw being about one inch, and that in the posterior triangle about two inches in diameter. There were tumors over the collar bone, the second rib, and the fourth costal cartilage near the sternum, each of which was in a direct line, and had followed in regular order the one described as existing in the posterior triangle. These tumors resemble each other in appearance; they are smooth and evenly formed, and are in shape as nearly as possible half a sphere; the upper one is two inches in diameter, the lower one an inch, and the middle one intermediate in size; they have an elastic, semi-fluctuating feel; the skin over the upper one is thin, red, and evidently about to open; the skin of the middle one is also discolored, that of the lowest is normal. To the right and left of these tumors are two nodules about the size of a marble, apparently the same thing in process of formation. The discharge was thin and serous, and contained minute yellowish masses, and disintegrated tissue, and had a peculiarly offensive and sour smell. The patient declined operative interference. He remained in the hospital till December 7th. The progress of the case being gradual loss of flesh, formation of lumps on the other side of the neck and in the axilla. The appearance of the tumors resembled nothing I had ever seen before. The case was certainly not scrofulous; nor was it like any new-growth with which I was familiar. I arrived at the conclusion that it was an example of the disease known as actinomycosis. This diagnosis was confirmed by the discovery under the microscope of bodies which I believed to resemble the fungus described as peculiar to this disease. So far as I can ascertain this is the first case of actinomycosis described in this country."

## Figures and Tables

**Fig. 1. f1:**
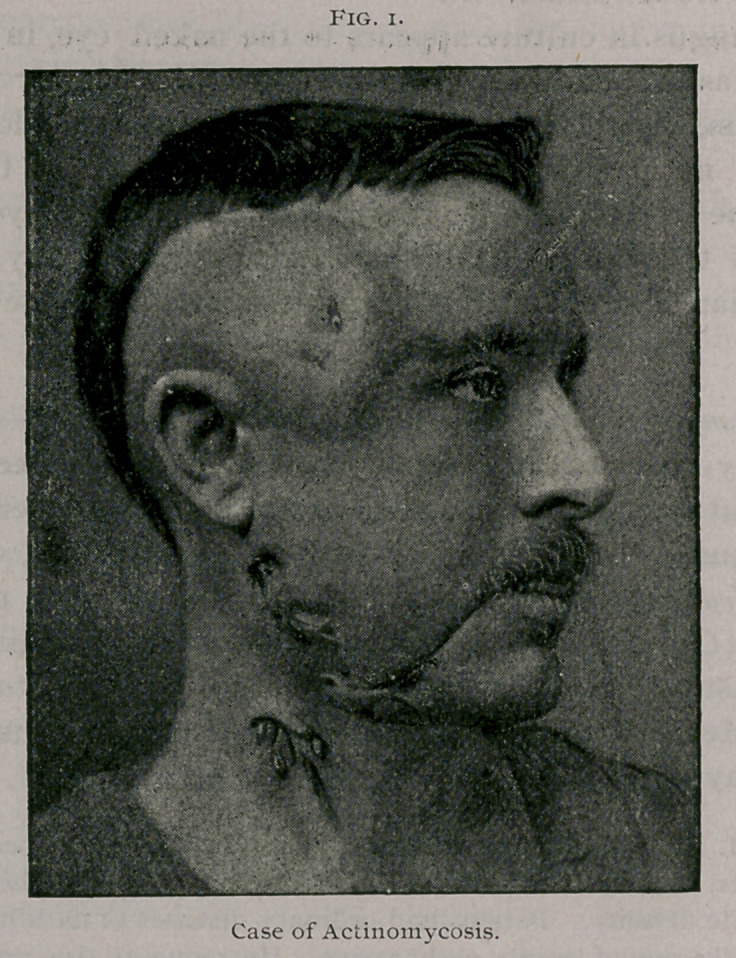


**Fig. 2. f2:**